# A systematic review of global mental health service utilisation in young refugees and asylum seekers

**DOI:** 10.1192/bjo.2025.10963

**Published:** 2026-03-02

**Authors:** Nada Abou Seif, Brian C. F. Ching, Jo Billings, Angeliki Argyriou, Victoria Pile, Patrick Smith

**Affiliations:** Institute of Psychiatry, Psychology, & Neuroscience, Kings College London, London, UK; Division of Psychiatry, University College London, London, UK; Helen Bamber Foundation, London, UK

**Keywords:** Refugees, asylum seekers, young people, service use, systematic review

## Abstract

**Background:**

Despite the high prevalence of mental health difficulties in young refugees and asylum seekers, evidence suggests that they underutilise mental health services. It is important that we understand their use of, and access to, mental health services.

**Aims:**

To examine quantitative evidence on mental health service utilisation and access among young refugees and asylum seekers.

**Method:**

We searched MEDLINE, PsycINFO, Embase, Global Health and The International Bibliography of the Social Sciences. Searches were supplemented by reference list screening and forward-and-backward citation tracking of included studies. Results were synthesised narratively. Our review was pre-registered on PROSPERO (no. CRD42024540885) and followed Preferred Reporting Items for Systematic Reviews and Meta-Analyses guidelines.

**Results:**

Twenty-two studies were included. We found an overall pattern of underutilisation of services by young refugees in comparison with majority population peers, particularly for out-patient services and psychotropic medication. In contrast, there was evidence of increased emergency service use. Service use was particularly decreased for those from low- and middle-income countries, and increased in unaccompanied minors. Service use for trauma-related disorders and schizophrenia was most common, and less likely for neurodevelopmental disorders. Only one study contained data on access-related factors, which identified language as a potential barrier.

**Conclusions:**

There is a disparity between the mental health needs and service use of young refugees, suggesting a need for greater efforts to increase access and use in this population. Future research should explore barriers and facilitators, and build on primary research examining service use in asylum seekers and unaccompanied minors, because both remain underrepresented within the literature.

There has been a rapid increase in the number of refugees and asylum seekers worldwide, with a 7% year-on-year increase in 2023 alone.^1^ As of June 2024, there were 43.7 million refugees worldwide and 1.9 million new asylum claims made.^2^ Young refugees and asylum seekers comprise approximately 40% of the global refugee and asylum-seeking population.^2^

## The mental health of young refugees and asylum seekers

Pre-displacement and asylum experiences often include persecution, war and other interpersonal traumatic events,^3–5^ making refugees and asylum seekers at particular risk for mental health difficulties. They then face additional post-migration stressors, such as uncertainty about legal status, language barriers, stigma and discrimination, separation from and worry about family members, financial instability and limited social support.^4,6,7^ Stressors of this nature are associated with mental health difficulties in general.^8–10^ Prevalence estimates in young refugees and asylum seekers have demonstrated elevated rates of post-traumatic stress disorder (PTSD), depression, anxiety disorders and emotional and behavioural problems.^11–14^ For example, one systematic review looking at the prevalence of these mental health difficulties in asylum-seeking children and adolescents reported high but widely varying rates of PTSD (interquartile range (IQR) 19.0–52.7%), depression (IQR 10.3–32.8%) and anxiety disorders (IQR 8.7–31.6%).^11^

## Mental health service utilisation among young refugees and asylum seekers

In comparison with non-refugee counterparts, young refugees underutilise mental health services irrespective of service type or level of care.^15–17^ A crucial first step in meeting the mental health needs of this population is to develop a comprehensive understanding of their utilisation of, and access to, mental health services, including any barriers or facilitators to access. This is particularly important for young refugees and asylum seekers, given the likelihood of linguistic barriers, differing conceptualisations of mental health and limited awareness of the availability of services and how to access them.

## The existing literature

Existing reviews have reported an underutilisation of mental health services by both young^18^ and adult refugees and asylum seekers.^19,20^ Stigma, structural barriers (e.g. language, lack of awareness of services, unstable accommodation) and refugee-specific barriers (e.g. immigration status) have been highlighted as likely barriers.^18–20^ However, most studies included in these reviews were conducted in Europe and the USA and therefore may not reflect global patterns. Additionally, the only review looking specifically at young refugees and asylum seekers^18^ was conducted 15 years ago, and an update is now needed. Evidence suggests that young people are particularly unlikely to access mental health services^21–23^ despite 75% of mental health difficulties occurring by age 24.^24^ Suggested reasons include stigma, a lack of mental health literacy and inaccessibility of services.^22^ This discrepancy between mental health needs and service use is particularly concerning given the associations among mental health difficulties and poor functioning, decreased educational attainment and social isolation in the long term.^25,26^

## Aims

We sought to examine recent quantitative evidence on mental health service use and access among young refugees and asylum seekers worldwide. Our specific aims were to:

- explore mental health service use in young refugees and asylum seekers;
- explore barriers and facilitators to accessing mental health services in young refugees and asylum seekers.

## Method

Our systematic review followed Preferred Reporting Items for Systematic Reviews and Meta-Analyses (PRISMA) guidelines^27^ and was pre-registered on the PROSPERO international prospective register of systematic reviews (no. CRD42024540885). A PRISMA checklist can be found in Supplementary Material A available at https://doi.org/10.1192/bjo.2025.10963. This review was completed as part of the first author’s doctoral thesis.^28^

### Search strategy and selection criteria

We conducted searches on Embase, Global Health, The International Bibliography of the Social Sciences, Ovid MEDLINE(R) ALL and APA PsycINFO. This allowed us to cover a broad range of clinical evidence across psychology, psychiatry, global health and the social sciences. The search was first conducted in May 2024 and updated in May 2025. Searches contained keywords pertaining to the relevant population (young refugees and asylum seekers), intervention (mental health services) and outcome (utilisation, access and barriers). Full search strategies for each database are available in Supplementary Material B. Searches were supplemented by reference list screening and forward- and backward-citation tracking of included studies.

Studies were included if they:

- explored mental health service utilisation, access or barriers;
- included samples of young refugees or asylum seekers (up to age 24 years, or median age of sample up to 24). This age criterion was informed by the United Nations Human Rights definition of youth refugees, which includes those up to age 24, as well as United Nations and WHO definitions of ‘youth’, which both include young people up to the age of 24 years;
- used quantitative methods;
- were published in peer-reviewed journals, to ensure rigour of evidence;
- were published in English;
- were published after April 2014, to capture current evidence.

We did not set any restriction on study design, presence of comparator groups or the nature or type of mental health service. Where applicable, if studies did not meet the age requirement but had extractable data for young refugees, these were still included. Qualitative studies were excluded, because the purpose of the review was to examine measurable patterns of health service use and quantifiable factors influencing access to mental health services among young refugees and asylum seekers.

### Study screening

Citations and abstracts of all search results were downloaded and exported to EndNote, version 21 for Windows (Clarivate, Philadelphia, Pennsylvania, USA; https://endnote.com/). All titles and abstracts were screened against inclusion criteria by the primary researcher (N.A.S.), and an independent reviewer (B.C.F.C.) screened a random sample of 20% of the articles. All (100%) full-text screening was replicated by the independent reviewer (B.C.F.C.). Interrater agreement was high for both title and abstract (97.6%, *κ* = 0.70) and full-text (94%, *κ* = 0.80) screening. All disagreements were resolved following discussion between the two reviewers.

### Data extraction

The following information was extracted from each included study: (a) authors, (b) year of publication, (c) country, (d) study aim, (e) study design and sample size, (f) population, (g) mental health outcome, (h) case determination method, (i) nature of mental health service being utilised, (j) service utilisation rate, (k) elements of access, (l) barriers and facilitators and (m) key findings. All (100%) data extraction was conducted by the primary researcher (N.A.S.) and replicated by the independent reviewer (B.C.F.C.). Data extraction for studies identified from the initial and updated searches began in August 2024 and May 2025, respectively. Interreviewer agreement was high (87.4%), and all disagreements were resolved following discussion between the two reviewers.

To answer our first review question, we conceptualised service utilisation as an estimate of contact coverage.^29^ This refers to the number of individuals who receive an intervention relative to the estimated number of individuals in the population who need an intervention. We did not seek to explore effective coverage, which looks at whether the desired health benefits have been achieved, because that was not the aim of our review and would probably include different literature.

To answer our second review question, we were guided by Penchansky and Thomas’ framework theory of access.^30^ This framework examines the following domains of access: affordability, availability, accessibility, accommodation and acceptability. In line with similar reviews,^19^ we sought to include stigma and awareness as factors impacting access.

### Quality appraisal

All (100%) quality appraisal was conducted by two independent reviewers (N.A.S. and B.C.F.C.) using the Joanna Briggs Institute (JBI) checklists for cohort studies and cross-sectional studies.^31^ This is a deviation from the protocol, which specified that quality would be appraised using the Effective Public Health Practice Project Quality Assessment Tool.^32^ We chose to use the JBI checklists because these are tailored to the specific study designs included within our review, allowing for a better assessment of potential bias. Checklists contain a total of 11 and 8 questions, respectively, examining sampling methods, measurement of exposure and outcome, management of confounders and the use of appropriate statistical analyses. Each item is rated as either ‘yes’, ‘no’, ‘unclear’ or ‘not applicable’. Studies were categorised as high quality if they answered ‘yes’ on 81–100% of items, moderate if they answered ‘yes’ on 51–80% of items and low if they answered ‘yes’ on 0–50% of items. Interreviewer agreement was high (90.86%, *κ* = 0.79). All disagreements were resolved following discussion between the two reviewers.

### Data analysis and synthesis

Having anticipated heterogeneity within studies, we used a narrative synthesis to summarise findings relevant to our review questions. We begin by synthesising findings on mental health service utilisation to answer our first review question. For each service type, we first present overall use, followed by use according to presenting difficulty. Within this, we explore any differences related to demographic- (e.g. age or gender) or refugee-specific factors (e.g. country of origin or parental presence). We then synthesise findings around facilitators and barriers to access to answer our second review question.

## Results

### Search results

**Fig. 1:**
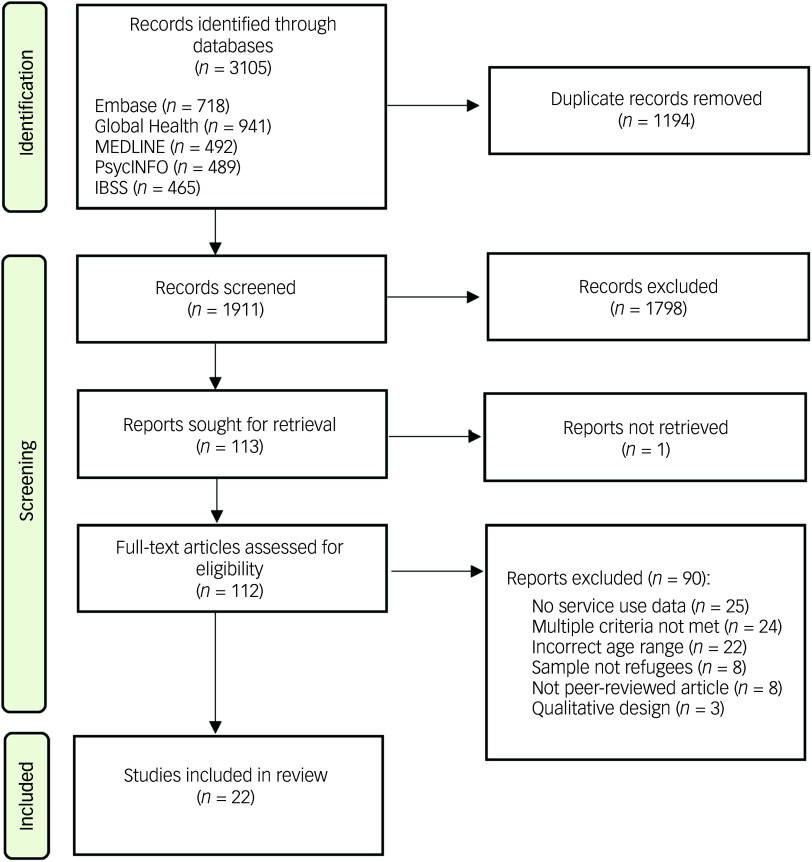
Preferred Reporting Items for Systematic Reviews and Meta-Analyses flow diagram. IBSS, International Bibliography of the Social Sciences.

Our search yielded 3105 records (Fig. 1). Following removal of duplicates, we screened the titles and abstracts of a total of 1911 records, of which 112 full-text articles were assessed for eligibility. A total of 22 studies were included in the final analysis.

### Study characteristics

An overview of study characteristics is shown in Supplementary Tables 1 (cohort studies) and 2 (cross-sectional studies). Included studies were conducted in Canada (*n* = 8), Sweden (*n* = 6), Denmark (*n* = 2), Turkey (*n* = 2), Australia (*n* = 1) and the USA (*n* = 1); two studies collected data from several low- and middle-income countries (*n* = 2). Of the included studies, 18 used a cohort design and 4 were cross-sectional. Data sources were either registers (*n* = 15) or clinical records (*n* = 7).

In line with our inclusion criteria and review focus, all studies reported on mental health service use by young refugee or asylum seekers. Importantly, all included studies looked only at young refugees (i.e. youth who had been granted residency permits) and not asylum seekers (i.e. pending asylum decision). As such, our findings reflect service use in young refugees and not asylum seekers. Studies looked at refugees who were either exclusively under 18 years old (*n* = 13), up to 25 years old (*n* = 8) or exclusively between 19 and 25 years old (*n* = 1). The number of refugees included in the studies ranged from 60^33^ to 41 884^34^. Studies compared service use with the majority population (*n* = 17) and/or other migrant youth (*n* = 3), or had no comparator (*n* = 4). For our synthesis, we chose to present results that have been adjusted for relevant factors (e.g. demographic and socioeconomic factors) whenever possible.

### Quality of included studies

Of the 18 cohort studies included, 11 were rated as high quality,^15–17,34–41^ 4 as moderate quality^33,42–44^ and 3 as low quality.^45–47^ Moderate- and low-quality studies did not adequately address items assessing confounding and follow-up. Two^48,49^ of the four cross-sectional studies were considered high quality, adequately addressing all aspects of the JBI appraisal checklist. The remaining two studies^50,51^ were assessed as being of moderate quality, as they did not provide sufficient information on whether confounders were identified and addressed. No studies were excluded based on quality, because the quality of existing literature is an important finding in itself. Full details of the quality appraisal can be found in Supplementary Material C.

### Q1. Mental health service utilisation

Of the 22 studies that looked at mental health service utilisation, half (*n* = 11) covered multiple healthcare service types and half (*n* = 11) reported only one service. Out-patient services were the most frequently reported (*n* = 11), followed by emergency services (*n* = 7), in-patient (*n* = 6), combined service types (*n* = 5), psychotropic medication prescription or use (*n* = 4) and primary care (*n* = 3). Almost half of the studies (*n* = 10) defined mental health service use as first contact or initiation of medication, with the remaining half looking at rates or amount of use. In addition to overall rates of service use, several studies sought to understand other factors that might influence service use, including the presenting difficulty/diagnosis (*n* = 10), country or region of origin (*n* = 5) and parental presence (*n* = 3).

### Combined service types

Six studies reported mental health service use as a variable that combined several types of services and compared refugee service use with that of the majority population. Studies looked at refugees who were exclusively under 18 years old,^36,37,42^ those aged up to 25 years^17,34^ and those exclusively between 19 and 25 years.^38^ Overall, these studies showed significantly lower service use in refugees in comparison with their Swedish, Danish and Canadian counterparts. For example, Kamali et al^42^ found that young refugees were 36% less likely than their Ontario-born peers to have had any mental health service contact (adjusted odds ratio (aOR) 0.64, 95% CI 0.58–0.71). Similarly, Amin et al^34^ found that young refugees had a 25% lower likelihood of being treated for common mental health difficulties in comparison with their Swedish counterparts (adjusted hazard ratio (aHR) 0.75, 95% CI 0.73–0.77). Only one study reported overall combined mental health service use in unaccompanied and accompanied minors separately, finding a lower likelihood of service use for both in comparison with Swedish-born individuals (unaccompanied minors aHR 0.65, 95% CI 0.58–0.72; accompanied minors aHR 0.75, 95% CI 0.72–0.78).^38^

The three studies that investigated country or region of origin reported that refugees from most regions of origin exhibited decreased use of mental health services in comparison with those belonging to the majority population.^34,36,37^ For example, Berg et al^37^ reported that young refugees from low- and middle-income countries (aHR 0.34, 95% CI 0.28–0.42; aHR 0.51, 95% CI 0.46–0.56, respectively) had significantly lower service use rates, whereas those from high-income countries did not differ from the majority population (aHR 0.92, 95% CI 0.70–1.21). Two studies also reported particularly low rates for young people from sub-Saharan Africa,^36^ the Horn of Africa^34^ and Syria.^34^ One exception to this trend was reported by Amin et al,^34^ in which young refugees from Iran demonstrated a slightly higher likelihood of being treated for common mental health disorders than majority peers (aHR 1.15, CI 1.05–1.26).

Only one study looked at the potential impact of age of arrival on mental health service use. Björkenstam et al^38^ reported that those that had arrived in Sweden over the age of 16 years had a 40% less likelihood of service use compared with the majority population (aHR 0.60, 95% CI 0.53–0.67), whereas those that arrived before age 7 years had only a 20% reduced likelihood (aHR 0.81, 95% CI 0.77–0.85).

Of the three studies looking at the influence of time since immigration, two found that care use increased over time of residence in Sweden.^37,38^ Berg et al^37^ reported that this was particularly evident for those who had received residency on the grounds of asylum (aHR <5 years: 0.59, 95% CI 0.48–0.72; aHR 10+ years: 0.74, 95% CI 0.64–0.86) rather than family reunification (aHR <5 years: 0.25, 95% CI 0.17–0.36; aHR 10+ years: 0.37, 95% CI 0.28–0.50). The remaining study found that use was highest in the first 2 years in Sweden (aOR 2.30, 95% CI 2.09–2.54), and that this was largely driven by unaccompanied refugee minors (aOR 3.39, 95% CI 2.96–3.85), although rates were still elevated in accompanied refugee minors (aOR 1.51, 95% CI 1.31–1.79).^17^ After 6 years, use for both became significantly lower than that for Swedish youth and, at 12 years, was no longer significant.^17^ Notably, this study adjusted only for gender and parental income, whereas the other two also adjusted for age.

All six studies looking at overall service use also explored use according to presenting difficulty. Young refugees generally had lower service use rates for most disorders when compared with majority youth, with this being particularly evident for neurodevelopmental disorders and difficulties related to substance use.^17,33,38^ For example, de Montgomery et al^16^ found a significantly lower likelihood of any contact for neurodevelopmental disorders and drug-related contact for both refugee boys (neurodevelopmental aOR 0.03–0.15; drug-related aORs 0.29–0.7) and girls (neurodevelopmental aOR 0.11–0.16; drug-related aOR 0.23–0.40).

In contrast, three studies found significantly higher use for trauma-related difficulties in comparison with the majority population. Björkenstam et al^38^ found that this changed depending on time since immigration. They reported that refugees with <6 years of residency had a threefold likelihood of care use for PTSD (aHR 3.31, 95% CI 2.40–4.56), whereas those who had lived in Sweden for 10 years or more were only at 1.5-fold likelihood (aHR 1.52, 95% CI 1.25–1.85).^38^ Two studies also found significantly higher use in refugees for schizophrenia and other non-affective psychotic disorders.^16,38^ For example, Björkenstam et al^38^ reported that refugee immigrants had 1.5-fold likelihood of use in comparison with their Swedish peers (aHR 1.49, 95% CI 1.29–1.71).

### Out-patient services

Eleven studies reported on out-patient mental healthcare use. Nine of these compared service use with the majority population,^16,33,35,36,44,48,49,51^ two with non-refugee migrants^35,43^ and one conducted a within-group comparison.^50^ Studies included refugees who were exclusively under 18 years old^33,35,36,40,43,44,50,51^ and those aged up to 25 years old.^16,48,49^ Nine studies found significantly lower use in refugee youth. For example, both Barghadouch et al^36^ and de Montgomery et al^16^ reported decreased out-patient service use for refugee boys (adjusted rate ratio (aRR) 0.47, 95% CI 0.44–0.51; aOR 0.39, 95% CI 0.34–0.43) and girls (aRR 0.42, 95% CI 0.39–0.46; aOR 0.34, 95% CI 0.31–0.39). This appeared to be consistent across all regions of origin, but particularly for sub-Saharan African youth.^36^ Betancourt et al found no significant difference in clinic-based out-patient therapy but reported that, in comparison with US youth, refugees were significantly more likely to receive in-home counselling (11.8 *v*. 3.6%, *P* < 0.05).^33^

In contrast, Axelsson et al^35^ looked specifically at service use by unaccompanied refugee minors and reported significantly higher likelihood of out-patient service use in unaccompanied refugee minors (aHR 1.10, 95% CI 1.01–1.18) in comparison with both the majority population and accompanied migrant minors. They also looked at whether there was a difference in mean time from immigration to out-patient care for unaccompanied refugee minors versus accompanied migrant minors, finding that unaccompanied refugees had significantly lower mean times (1.37 *v*. 3.10 years, *P* < 0.001).^35^

Two of the above studies also reported specifically on private out-patient service use in comparison with Danish youth, with both reporting significantly lower use in both refugee boys and girls.^16,36^ For example, Barghadouch et al^36^ found aRRs of 0.25 (95% CI 0.22–0.28) and 0.16 (95% CI 0.14–0.18) in refugee boys and girls, respectively.^36^ Rates were low across all regions of origin, but particularly for sub-Saharan African children. De Montgomery et al^16^ also reported that the numbers of consultations with private practitioners were also lower in young refugees.

In addition, one study looking specifically at refugee mental health service use in the context of the COVID-19 pandemic reported a 20% increase (aRR 1.20) in mental health service visits for refugees that were new to care during the first year of the pandemic.^50^ This increase was not seen in those who had pre-existing and ongoing contact with services.^50^

Three studies reported on presenting difficulties in out-patient services. Both studies conducted in Turkey reported neurodevelopmental disorders as the most common presenting difficulty.^44,47^ For Karadag and Calisgan,^47^ 34% of refugee youth presenting to the service was diagnosed with an intellectual disability. For Poyraz Fındık et al,^44^ attention-deficit hyperactivity disorder (ADHD) was the most common presenting difficulty for both refugees and non-refugees. However, they reported that depression and PTSD were more common in refugee children, with all PTSD diagnoses in the service being in the refugee group. Notably, they also reported that the non-refugee group was seven times more likely to receive a diagnosis than the refugee group (1.2 *v*. 8.8% did not receive a diagnosis). However, both studies were rated poorly in our quality appraisal, and therefore their findings should be interpreted with caution.

The remaining study,^16^ conducted in Denmark, reported significantly fewer and shorter out-patient contacts for affective, neurotic and stress-related disorders in refugee girls compared with non-refugee girls, as well as for schizophrenic disorders in refugee boys compared with non-refugee boys.

### In-patient services

Six studies reported on in-patient service use in comparison with the majority population. Studies looked at refugees who were exclusively under 18 years old^33,35,36,40^ and those aged up to 25 years.^15,16^ Findings appeared to differ depending on where studies were conducted. All three studies conducted in North America reported no significant difference in service use when comparing young refugees with majority population youth,^15,33,40^ with Saunders et al^15^ also reporting comparable rates across all places of origin for refugees.

By contrast, two studies conducted in Denmark demonstrated decreased use of in-patient care among refugees.^16,36^ Both reported decreased likelihood of in-patient care for both refugee boys (aOR 0.59, 95% CI 0.50–0.70; aOR 0.59, 95% CI 0.43–0.85) and girls (aOR 0.46, 95% CI 0.39–0.54; aOR 0.50, 95% 0.47–0.85, respectively). Barghadouch et al^36^ also looked at the impact of place of origin and reported that, although rates for almost all regions were significantly lower, those for boys from the former Yugoslavia were particularly low (aRR 0.20, 95% CI 0.08–0.49). They also reported increased rates for children from sub-Saharan Africa, but notably these were not significant.

The remaining study investigated service use in unaccompanied refugee minors, and reported a higher likelihood of using in-patient care (aHR 1.30, 95% CI 1.10–1.45) in comparison with both the majority population and accompanied migrant minors.^35^ Looking at mean time from immigration to in-patient care, they found that unaccompanied refugees had significantly lower mean times in comparison with accompanied migrant minors (1.58 *v*. 3.86 years, *P* < 0.001).^35^

Only one study looked at the length of hospitalisation by presenting difficulty.^16^ In terms of duration of hospitalisation, it found that refugee boys had significantly longer hospital stays for affective disorders and schizophrenic disorders when compared with their Danish peers. In contrast, refugee girls had significantly shorter hospitalisations for affective disorders than their Danish peers.

### Emergency services

Seven studies reported on refugee mental health contact with emergency departments. Studies included refugees who were exclusively under 18 years old^33,36,40^ and those aged up to 25 years old.^15,16,39,49^ Findings differed depending on where studies were conducted. All four studies conducted in Ontario, Canada reported greater risks or rates of emergency room contact.^15,39,40,49^ For example, Saunders et al^15^ found significantly higher rates of first mental health contact in emergency departments among refugees in comparison with the majority population (aRR 1.17, 95% CI 1.13–1.21).

In contrast, the two studies conducted in Denmark found decreased rates among both refugee boys and girls.^16,36^ For example, de Montgomery et al^16^ reported decreased likelihood of first-time emergency room contact in refugee boys (aOR 0.65, 95% CI 0.55–0.76) and girls (aOR 0.49, 95% CI 0.42–0.57), but noted that this was significantly higher in refugee boys presenting with schizophrenia (aOR 1.61, 95% CI 1.22–2.21). Barghadouch et al^36^ also considered place of origin, and reported significantly lower rates in youth from the former Yugoslavia (risk ratio girls 0.37, 95% CI 0.28–0.49; risk ratio boys 0.27, 95% CI 0.17–0.41) and girls from sub-Saharan Africa (risk ratio 0.45, 95% CI 0.30–0.68), but significantly higher rates for girls from the Middle East and North Africa (risk ratio 1.41, 95% CI 1.20–1.65). The remaining study, conducted in the USA, found no significant differences in emergency room contact.^33^

Two studies looked at emergency room use by presenting difficulty. Saunders et al^49^ looked at first-contact emergency department visits and reported a higher proportion of visits in refugees versus Ontario-born youth for substance-related disorders (74.5 *v*. 66.8%), residual self-harm (69.8 *v*. 50.8%), acute stress (62.1 *v*. 47.5%) and anxiety (57.3 *v*. 50.4%). Rates were similar only for mood/affective disorder (38.8 *v*. 38%) and psychotic disorders (43.1 *v*. 40.7%). De Montgomery et al^16^ looked at the number of emergency room visits and found significantly greater levels for affective disorders in both refugee girls and boys in comparison with their Danish counterparts. They also reported that refugee girls had signifintly more emergency room visits for neurotic and stress-related disorders.

### Primary care

Three studies reported refugee primary care mental health contact. All three included only those refugees who were exclusively under the age of 18 years.^33,45,46^ Only one of these studies compared refugees with the majority population, and found no significant difference between refugees and US youth; however, it did report that refugee immigrants were more likely than non-refugees to use primary care (23.3 *v*. 5.8%, *P* < 0.05).^33^ The remaining two studies utilised the same data-set to look at service use within refugee primary healthcare facilities.^45,46^ They found that service use rates per 1000 per month were lower among children <5 years compared with refugees aged 5 years and above,^45,46^ and were higher among boys (weighted mean rate 1.13, s.d. 1.75) than girls (weighted mean rate 0.80, s.d. 1.20).^45^ Notably, both studies were rated as poor during quality appraisal, which should be kept in mind when considering their findings.

Two studies reported mean rates of service use in refugee primary healthcare facilities by presenting difficulty/diagnosis. Because Fine et al^46^ is an update of Kane et al,^45^ only the results of the former^46^ will be presented. They found that, for both boys and girls under 5 years, the highest rates of visits during the study period were for ‘epilepsy/seizure’, with rates per 1000 per month ranging from 0.33 to 0.59.^46^ This was followed by intellectual disability, with rates per 1000 per month ranging from 0.02 to 0.11.^46^ Service use rates for all other presenting difficulties/diagnoses were negligible.^46^

### Medication

Four studies looked at psychotropic medication use in young refugees, those who were exclusively under 18 years old^35,47^ and those aged up to 25 years old.^16,41^ Three studies found that refugees had lower medication prescription and use across gender and most presenting difficulties.^16^ For example, Taipale et al^41^ found young refugees had 57% reduced odds of initiating antidepressants in comparison with their Swedish peers (aOR 0.43, 95% CI 0.39–0.58), and that this was consistent across youth seen for depression, anxiety and PTSD (aOR 0.42–0.49). This appeared to vary slightly according to country of origin, with refugee youth from Somalia (odds ratio 0.70) and Iraq (odds ratio 0.79) being less likely to initiate antidepressant use in comparison with those from the former Yugoslavia. Notably, refugees also more frequently had a lag time >7 days between prescription and dispensing of antidepressants (16.8 *v*. 8.6%). Taipale et al^41^ also reported that having fewer than 5 years of residency in Sweden was associated with decreased initiation of antidepressants (odds ratio 0.76, 95% CI 0.63–0.92), and older age was associated with increased initiation (odds ratio 1.07, 95% CI 1.04–1.10).

The remaining study found that unaccompanied refugee minors (aHR 1.10, 95% CI 1.01–1.19) were more likely to be prescribed medication in comparison with their Swedish peers.^35^ Unaccompanied refugee minors also had significantly lower mean time to first prescribed medication in comparison with accompanied migrant minors (1.58 *v*. 4.47 years, *P* < 0.001).^35^

Three studies looked at whether this differed by type of medication. Taipale at al^41^ reported that, alongside decreased antidepressant initiation, young refugees were also less likely to be started on other psychopharmacotherapy (51.5 *v*. 61.8%), particularly anxiolytics (52.3 *v*. 60%). They also found that hypnotics were more commonly prescribed (34.8 *v*. 32.8%).^41^ Karadag et al^47^ reported that an equal proportion of refugee children was prescribed selective serotonin reuptake inhibitors (SSRIs) and antipsychotics (16 and 17%, respectively). Axelsson et al^35^ reported that, in comparison with their Swedish peers, unaccompanied refugee minors had a higher likelihood of being prescribed all psychotropic medication (aOR 1.27–3.25), except for ADHD medication (aOR 0.06, 95% CI 0.04–0.10). This was particularly pronounced in regard to sedatives, with unaccompanied minors having over three times the likelihood of prescription (aOR 3.25, 95% CI 3.01–3.51).^35^

### Q2. Access to mental health services

Only one included study looked at factors associated with access to mental health services, and highlighted language ability as a potential barrier to service use.^43^ Mohamud et al^43^ found that refugees were significantly less likely to use virtual mental health visits in comparison with economic immigrants, regardless of their Canadian language ability. However, refugees with non-Canadian language ability had a 9% lower likelihood of virtual care utilisation compared with refugees with Canadian language ability.

## Discussion

### Main findings

The main finding of our review was an overall pattern of underutilisation of mental health services among young refugees. This is in line with existing reviews^18–20^ but in stark contrast to established elevated rates of mental health difficulties.^11,52,53^ On an individual level, it may be that young refugees have different conceptualisations of mental health difficulties, viewing them through the lens of social, cultural, religious and political difficulties.^54^ In line with this, evidence suggests that young refugees and asylum seekers prefer informal support^54,55^ and are more likely to seek peer-, community- and religious-based support.^56,57^ Additional individual-level barriers include limited knowledge of mental healthcare systems, language skills and stigma among both refugees and asylum seekers themselves, and their parents or families.^54,56,58,59^ This is also aligned with our finding of increased service use with longer duration of residency. As young refugees and asylum seekers become more accustomed to their host countries, they are likely to overcome the aforementioned barriers.^60^ It may also be that, with time, young refugees’ and asylum seekers’ legal status and other post-migration stressors are settled, allowing greater capacity to acknowledge mental health needs and seek relevant support.

At the system level, a lack of cultural understanding and competence within services may prohibit the adequate identification of mental health difficulties among young refugees and asylum seekers. It may be that commonly drawn-upon conceptualisations of mental health difficulties (e.g. diagnostic criteria) are not culturally sensitive to the presentations of this population. For example, Yim et al^61^ described social worker concerns about the rejection of referrals to child and adolescent mental health services for unaccompanied minors due to their expression of distress not fitting into Western diagnostic frameworks. There may also be a tendency to dismiss the mental health symptoms of young refugees and, instead, attribute their difficulties to migration-related or social issues. Structural barriers such as distance, unstable housing and a lack of resources may also play a role.^54,62^

Our finding that young refugees are more likely to seek mental healthcare through emergency departments suggests a crisis-driven approach. Attendance at emergency departments usually reflects greater clinical need, which may indicate delayed or inadequate recognition of mental health difficulties in young refugees and asylum seekers, particularly when viewed alongside the finding of reduced out-patient service use. This may be explained by a lack of awareness of mental healthcare services, preference for seeking care in hospital or young refugees’ and healthcare providers’ inability to recognise the mental health needs of this group until the point of crisis. It may also reflect deprivation, as there is evidence that youth from deprived neighbourhoods are more likely to have their first contact in an emergency department.^63^ Notably, we identified differences in this finding between North American and Danish studies, which may reflect their respective healthcare systems and the support structures in place for young refugees. The largely privatised healthcare system in the USA means that refugees face delayed or limited access to mental healthcare due to insurance ineligibility.^64,65^ Similarly, while Canada offers universal healthcare, the Interim Federal Health Program provides only temporary and limited healthcare coverage.^66^ While this includes some mental health services, eligibility criteria, referral requirements and session limits may act as barriers to access for refugees. In contrast, Denmark’s universal healthcare system places more emphasis on comprehensive integration of mental health services, including community- and school-based programmes, which may facilitate earlier and preventive care.^67–69^ However, it is important to note that such findings are primarily relevant to older adolescents and young adults, for whom psychiatric emergencies (e.g. self-harm, acute psychosis) are more prevalent. These may not reflect patterns of emergency service use in younger children, as evidence suggests that mental health-related emergency visits are particularly uncommon in this age group.^70^

We also found that young refugees from low- and middle-income countries demonstrated decreased use, whereas those from high-income countries were comparable to the majority population. Similar patterns have been seen in young, non-refugee immigrants.^71^ There is also evidence that children with parents from low-income countries tend to utilise mental health services less than those with native-born parents.^72,73^ This may be explained by a lack of familiarity with mental healthcare systems due to differing mental health conceptualisations and healthcare systems in countries of origin.^74–76^ This is likely to be particularly evident in those from low-income countries, as the World Health Organization^76^ has highlighted a lack of youth psychiatric services in these nations. Additionally, limited resources within low-income countries may compel governments to prioritise fundamental needs (e.g. food and housing) at the expense of mental healthcare, potentially resulting in limited exposure to, and familiarity with, mental health services prior to resettlement. This lack of familiarity, alongside potential stigma surrounding mental health difficulties among certain cultural groups originating from these nations, may further inhibit service use among young refugees. In contrast, our finding of increased service use among young Iranian refugees can be explained by Iran’s robust healthcare system, which has integrated mental health treatment within the primary care system in an effort to increase access to care.^77,78^

Findings on service use among unaccompanied refugee minors are mixed. This may be explained by a difference in age parameters set within studies exploring this. Björkenstam et al^38^ included youth who were aged 19–25, but had arrived in Sweden as unaccompanied minors, and reported significantly decreased use among both unaccompanied and accompanied minors. In contrast, Axelsson et al^35^ and Gubi et al^17^ included only youth who were minors and reported significantly increased use among unaccompanied minors. Nonetheless, increased service use among unaccompanied refugee minors is expected given their increased and persistent mental health needs.^53,79–82^ For example, Müller et al^53^ reported that prevalence rates of PTSD, depression and anxiety were twice as high among unaccompanied youth in comparison with accompanied counterparts. It may also be that greater involvement of unaccompanied youth with social care facilitates access to mental health services. All studies that included unaccompanied minors were conducted in Sweden, where unaccompanied minors are assigned mandated guardians.^83^ Mandated guardians can bypass the barriers to care that may be experienced by young refugees and asylum seekers (e.g. proficiency in navigating healthcare systems and language).

Service use also varied by presenting difficulty. Young refugees’ use of mental health services primarily for PTSD and other stress-related difficulties is expected, given the high prevalence of PTSD within this population.^11,52,84^ However, the lack of use for depression and anxiety is notable, given that these are also highly prevalent among young refugees.^84,85^ The distinct lack of service use for neurodevelopmental disorders is also worth noting. This may be attributable to diagnostic overshadowing, with clinicians either missing or being reluctant to diagnose neurodevelopmental disorders or other/comorbid mental health difficulties. Biases may also be playing a role, with young refugees and asylum seekers not being offered treatment for anxiety and depression due to clinicians conceptualising their difficulties as more rooted in migration-related or social stressors and traumas. Another explanation is that cultural variation in presentation and understanding of symptoms is contributing to misdiagnoses or missed diagnoses.^86^ Furthermore, young refugees are likely to be experiencing an ongoing process of acculturation, including learning a new language and adapting to unfamiliar social and cultural norms.^60^ In turn, this may create further challenges with assessment, particularly in relation to reciprocal social communication and adaptive functioning, subsequently increasing the risk of missed diagnoses.

Our review builds on existing evidence of the decreased use of psychotropic medication among adult refugees.^87^ Notably, we found that young refugees were less likely than their Swedish counterparts to initiate the use of SSRIs, which are the first-line medication for depression,^88^ and were more commonly prescribed sedative-hypnotics. Sedative-hypnotics are often used to treat sleep difficulties, which are common among young refugees and asylum seekers.^89,90^ This may reflect young refugee preference and a desire to address a specific need (i.e. sleep disturbance). Alternatively, it may be that there is a tendency for clinicians to focus on PTSD or social stressors, in which case drug treatments would not be offered to those under 18 years.^91^ Instead, National Institute for Health and Care Excellence guidance states that young refugees and asylum seekers should be offered individual trauma-focused therapy.^91^ However, our findings suggest that this is also not being offered. An additional explanation may be lack of financial ability, given that studies examining medication use were conducted in Sweden and Denmark, where individuals need to at least partially pay for medication.

### Implications

This review has highlighted the need to facilitate access along the mental healthcare pathway. Young refugees and asylum seekers should be screened for mental health difficulties in a timely and adequate manner, and efforts should be made to address potential barriers to access. Within this, further education for young refugees and asylum seekers, their families and healthcare professionals is pivotal. Young refugees and asylum seekers and their networks should be supported to develop an understanding of mental healthcare systems within their host country. They should also be supported in fostering greater mental health literacy to aid in the reduction of associated stigma. Importantly, the aim here should not be to force certain conceptualisations of mental health onto young refugees and asylum seekers, but simply to empower them to navigate the systems of which they are now a part.

Alongside this, healthcare professionals should be supported to develop greater cultural awareness and understanding of mental health presentations in young refugees and asylum seekers. The potential for biases or shying away from diagnosing certain presentation (e.g. neurodevelopmental diagnoses) should be explicitly addressed. To address language barriers, the use of interpreters and translated documents is encouraged. Services should ensure that they have access to skilled and trustworthy interpreters, and give young people choice over use of interpreters to allow trusting working relationships.

Given their increased vulnerability, particular attention should be paid to unaccompanied refugee youth. It may be of benefit to replicate the Swedish system and mandate a guardian for unaccompanied minors. Young refugees and asylum seekers and their families would also benefit from a mandated ‘guide’ to aid them in navigating unfamiliar systems, and to advocate for their social, health and legal needs. Although this is likely to come at a considerable cost, it may be that it is offset by the potential cost-effectiveness of earlier and more appropriate use of mental health services within this population.

Finally, the interaction among refugees’ socioeconomic disadvantage, structural barriers and increased mental health difficulties cannot be ignored. The Inter-Agency Standing Committee (IASC) model of Mental Health and Psychosocial Support consists of a four-level ‘Intervention Pyramid’: basic services and security, community and family support, focused non-specialised support and specialised services.^92^ As per this model, basic services and security are the foundation of mental health and psychosocial support. There is a need for the development and implementation of policies ensuring that legal protection, appropriate and safe accommodation, resources, educational and integration opportunities are afforded to this vulnerable population.^92^ The introduction and evaluation of the potential benefits of interventions of this nature that encompass IASC’s layer of basic services and security would be of benefit.

### Strengths and limitations

To our knowledge, this is the first systematic review looking at global mental health service utilisation in young refugees and asylum seekers. We conducted an extensive search across a variety of databases, allowing us to cover a wide range of cross-disciplinary evidence. We did not set any restrictions on types of services or countries of publication, which provided insight into patterns of service use across different types of services, countries and healthcare systems. We also did not set any restrictions on refugee-specific factors, such as immigration status (e.g. asylum seekers or refugees), parental presence or country or region of origin. This allowed us gain insight into the literature on service use across a broad range of young refugees and asylum seekers, and particularly to identify gaps in the literature.

However, there are several limitations to consider. Our review was inherently limited by the included studies, which were heterogeneous in measurement, analysis and quality. Notably, only half (*n* = 13) of the studies were assessed as high quality. There was also a clear exclusion or limited inclusion of asylum seekers and unaccompanied refugees. No studies include asylum seekers, and only three considered the difference in service use in unaccompanied versus accompanied refugees. Given that asylum seekers are a particularly vulnerable group, this is another significant limitation of the literature. Additionally, most studies included broad age ranges (e.g. 0–17 or 10–24) and did not present results stratified by age. This limited our ability to examine potential variations in patterns of service use across developmental stages (e.g. early childhood versus adolescence versus young adulthood). Given the substantial developmental differences in morbidity profiles – such as the higher prevalence of developmental and behavioural disorders in younger children^93–96^ and the relative rarity of conditions such as psychosis, substance misuse or self-harm in these younger age groups,^96^ aggregating broad age ranges may obscure clinically meaningful variation in help-seeking and service utilisation. Similarly, studies that included mixed-age samples did not distinguish between whether young people accessed child/adolescent or adult services. Although findings were generally consistent across studies regardless of age composition when looking at patterns of service use by service type, we acknowledge that this limits the extent to which our conclusions can speak to service-specific patterns. As such, the implications of differing service structures, thresholds and referral pathways cannot be fully assessed, and our results should be interpreted as reflecting general patterns across broadly defined ‘young refugees’.

There were also limitations of the review itself. Our searches were limited to the English language, which may have excluded relevant literature published in other languages and skewed our results to represent service use in primarily high-income countries, specifically in Europe and North America. The exclusion of grey literature may have led us to miss relevant evidence from charities working with young refugees and asylum seekers. Furthermore, only 20% of titles and abstracts were dual screened, which may have increased the risk of missed or incorrectly excluded studies. Our ability to explore access to services, including barriers and facilitators, was likely limited by our decision to include only quantitative literature. Given that qualitative methods are often better suited to answering questions seeking to understand experiences and perspectives,^97^ it may be that qualitative literature is better suited to answer this question.

### Future research

The main gaps we identified were studies looking at differences in mental health service utilisation based on immigration status (i.e. refugee versus asylum seeker) and parental presence (i.e. unaccompanied versus accompanied). Future research should seek to fill this gap to establish a more nuanced understanding of the needs of this population. Future research should also utilise age-stratified analyses and a clear delineation of child/adolescent versus adult mental health service use to better understand developmental nuances in help-seeking among young refugees and asylum seekers. It may also be of benefit to conduct research evaluating the cost-effectiveness of earlier and more appropriate use of services in this population. A systematic review of qualitative literature exploring barriers and facilitators to service access would also be of benefit.

In conclusion, our review suggests an underutilisation of mental health services by young refugees in comparison with majority population peers, particularly for out-patient services and psychotropic medication. We found that this did not apply to unaccompanied refugees, who had an increased likelihood of accessing services in comparison with the majority population. Young refugees were more likely to use services for trauma-related disorders and schizophrenia, and less likely to use services for neurodevelopmental disorders. Although consistent patterns emerged across studies, these findings should not be taken to reflect developmental differences or to characterise service use within specific systems (e.g. child and adolescent psychiatry versus adult psychiatry). Instead, the conclusions reflect broad, system-level patterns across heterogeneous samples of young refugees. There is a need for further exploration of barriers and facilitators to accessing mental health services in this population, particularly for asylum seekers and unaccompanied refugee minors. To meet this need, a systematic review of qualitative evidence exploring barriers and facilitators to access is encouraged.

## Supporting information

Abou Seif et al. supplementary material 1 Abou Seif et al. supplementary material

Abou Seif et al. supplementary material 2 Abou Seif et al. supplementary material

## Data Availability

Data availability is not applicable to this article because no new data were created or analysed in this study.

## Supplementary material

The supplementary material is available online at https://doi.org/10.1192/bjo.2025.10963
