# Supplementary material for: A systematic review of global mental health service utilisation in young refugees and asylum seekers
Source: BJPsych Open. 2026 Mar 2;12(2):e76. doi: 10.1192/bjo.2025.10963 (PMC12963839; doi:10.1192/bjo.2025.10963)
Supplement: Abou Seif et al. supplementary material 1 — Abou Seif et al. supplementary material [file S2056472425109630sup001.pdf]

SUPPLEMENTARY MATERIAL

A. PRISMA Checklist

Section and Topic	#	Checklist item	Location
**TITLE**			
Title	1	Identify the report as a systematic review.	Title
**ABSTRACT**			
Structured summary	2	Provide a structured summary including, as applicable: background; objectives; data sources; study eligibility criteria, participants, and interventions; study appraisal and synthesis methods; results; limitations; conclusions and implications of key findings; systematic review registration number.	Abstract
**INTRODUCTION**			
Rationale	3	Describe the rationale for the review in the context of what is already known.	Introduction - The existing literature
Objectives	4	Provide an explicit statement of questions being addressed with reference to participants, interventions, comparisons, outcomes, and study design (PICOS).	Introduction - Aims
**METHODS**			
Protocol and registration	5	Indicate if a review protocol exists, if and where it can be accessed (e.g., Web address), and, if available, provide registration information including registration number.	Method
Eligibility criteria	6	Specify study characteristics (e.g., PICOS, length of follow-up) and report characteristics (e.g., years considered, language, publication status) used as criteria for eligibility, giving rationale.	Method – Search strategy and selection criteria

Section and Topic	#	Checklist item	Location
Information sources	7	Describe all information sources (e.g., databases with dates of coverage, contact with study authors to identify additional studies) in the search and date last searched.	Method – Search strategy and selection criteria
Search	8	Present full electronic search strategy for at least one database, including any limits used, such that it could be repeated.	Supplementary material A
Study selection	9	State the process for selecting studies (i.e., screening, eligibility, included in systematic review, and, if applicable, included in the meta-analysis).	Method – Search strategy and selection criteria & Study screening
Data collection process	10	Describe method of data extraction from reports (e.g., piloted forms, independently, in duplicate) and any processes for obtaining and confirming data from investigators.	Method – Data extraction
Data items	11	List and define all variables for which data were sought (e.g., PICOS, funding sources) and any assumptions and simplifications made.	Method – Search strategy and selection criteria & Data extraction
Risk of bias in individual studies	12	Describe methods used for assessing risk of bias of individual studies (including specification of whether this was done at the study or outcome level), and how this information is to be used in any data synthesis.	Method – Quality appraisal
Summary measures	13	State the principal summary measures (e.g., risk ratio, difference in means).	Method – Data extraction
Synthesis of results	14	Describe the methods of handling data and combining results of studies, if done, including measures of consistency (e.g., I^2) for each meta-analysis.	Method – Data analysis and synthesis

Section and Topic	#	Checklist item	Location
Risk of bias across studies	15	Specify any assessment of risk of bias that may affect the cumulative evidence (e.g., publication bias, selective reporting within studies).	N/A
Additional analyses	16	Describe methods of additional analyses (e.g., sensitivity or subgroup analyses, meta-regression), if done, indicating which were pre-specified.	N/A
**RESULTS**			
Study selection	17	Give numbers of studies screened, assessed for eligibility, and included in the review, with reasons for exclusions at each stage, ideally with a flow diagram.	6
Study characteristics	18	For each study, present characteristics for which data were extracted (e.g., study size, PICOS, follow-up period) and provide the citations.	Supplementary Tables 1 and 2
Risk of bias within studies	19	Present data on risk of bias of each study and, if available, any outcome level assessment (see item 12).	Supplementary material C
Results of individual studies	20	For all outcomes considered (benefits or harms), present, for each study: (a) simple summary data for each intervention group (b) effect estimates and confidence intervals, ideally with a forest plot.	Supplementary Tables 1 and 2
Synthesis of results	21	Present results of each meta-analysis done, including confidence intervals and measures of consistency.	Results
Risk of bias across studies	22	Present results of any assessment of risk of bias across studies (see Item 15).	N/A
Additional analysis	23	Give results of additional analyses, if done (e.g., sensitivity or subgroup analyses, meta-regression [see Item 16]).	N/A
**DISCUSSION**			
Summary of evidence	24	Summarize the main findings including the strength of evidence for each main outcome; consider their relevance to key groups (e.g., healthcare providers, users, and policy makers).	Discussion – Main findings
Limitations	25	Discuss limitations at study and outcome level (e.g., risk of bias), and at review-level (e.g., incomplete retrieval of identified research, reporting bias).	Discussion - limitations
Conclusions	26	Provide a general interpretation of the results in the context of other evidence, and implications for future research.	Discussion - conclusions

Section and Topic	#	Checklist item	Location
**FUNDING**			
Funding	27	Describe sources of funding for the systematic review and other support (e.g., supply of data); role of funders for the systematic review.	N/A

B. Search Strategies

MEDLINE

	Search term	Results
1	exp Young Adult/	1133194
2	exp Child/	2268324
3	exp Adolescent/	2328791
4	(young or youth or child* or adolescen* or teen* or "young adult*").mp. [mp=title, book title, abstract, original title, name of substance word, subject heading word, floating sub-heading word, keyword heading word, organism supplementary concept word, protocol supplementary concept word, rare disease supplementary concept word, unique identifier, synonyms, population supplementary concept word, anatomy supplementary concept word]	5012969
5	1 or 2 or 3 or 4	5012969
6	exp Refugees/	14702
7	(refugee* or "asylum seeker*" or "forced migrant*").mp. [mp=title, book title, abstract, original title, name of substance word, subject heading word, floating sub-heading word, keyword heading word, organism supplementary concept word, protocol supplementary concept word, rare disease supplementary concept word, unique identifier, synonyms, population supplementary concept word, anatomy supplementary concept word]	21539
8	6 or 7	21539
9	5 and 8	8704
10	("unaccompanied minor*" or "accompanied minor*").mp. [mp=title, book title, abstract, original title, name of substance word, subject heading word, floating sub-heading word, keyword heading word, organism supplementary concept word, protocol supplementary concept word, rare disease supplementary concept word, unique identifier, synonyms, population supplementary concept word, anatomy supplementary concept word]	243
11	9 or 10	8808
12	exp Mental Health Services/ (	110834
13	(("mental" or "psych*" or "behavio?r*" or "psychosocial") adj4 ("health care" or "service*" or "treatment*" or "support*" or "contact*")).mp. [mp=title, book title, abstract, original title, name of substance word, subject heading word, floating sub-heading word, keyword heading word, organism supplementary concept word, protocol supplementary concept word, rare disease supplementary concept word, unique identifier, synonyms, population supplementary concept word, anatomy supplementary concept word]	263012
14	12 or 13	308504
15	exp Help-Seeking Behavior/	1520

16	exp "Patient Acceptance of Health Care"/	246150
17	("help seeking" or "seek* help" or "treatment seek*" or "seek* treatment" or "help seeking behavio?r" or "utili?ation" or "use*" or "contact*").mp. [mp=title, book title, abstract, original title, name of substance word, subject heading word, floating sub-heading word, keyword heading word, organism supplementary concept word, protocol supplementary concept word, rare disease supplementary concept word, unique identifier, synonyms, population supplementary concept word, anatomy supplementary concept word]	12008340
18	15 or 16 or 17	12129416
19	(barrier* or hurdle or obstacle or obstruct* or refusal or imped* or promot* or facilitat* or support* or enabl* or cause* or reason*).mp. [mp=title, book title, abstract, original title, name of substance word, subject heading word, floating sub-heading word, keyword heading word, organism supplementary concept word, protocol supplementary concept word, rare disease supplementary concept word, unique identifier, synonyms, population supplementary concept word, anatomy supplementary concept word]	16095014
20	exp Health Services Accessibility/	144292
21	("access" or "affordability" or "availability" or "accessibility" or "accommodation" or "acceptability" or "stigma" or "awareness").mp. [mp=title, book title, abstract, original title, name of substance word, subject heading word, floating sub-heading word, keyword heading word, organism supplementary concept word, protocol supplementary concept word, rare disease supplementary concept word, unique identifier, synonyms, population supplementary concept word, anatomy supplementary concept word]	1278691
22	20 or 21	1325721
23	18 or 19 or 22	22257324
24	11 and 14 and 23	820
25	limit 24 to (english language and humans and yr="2014 -Current")	492

Embase

	Search term	Results
1	exp young adult/	608199
2	exp child/	3335256
3	exp adolescent/	1928755
4	(young or youth or child* or adolescen* or teen* or "young adult*").mp. [mp=title, book title, abstract, original title, name of substance word, subject heading word, floating sub-heading word, keyword heading word, organism supplementary concept word, protocol supplementary concept word, rare disease supplementary concept word, unique identifier, synonyms, population supplementary concept word, anatomy supplementary concept word]	5183533
5	1 or 2 or 3 or 4	5768952
6	exp refugee/	19907
7	(refugee* or "asylum seeker*" or "forced migrant*").mp. [mp=title, book title, abstract, original title, name of substance word, subject heading word, floating sub-heading word, keyword heading word, organism supplementary concept word, protocol supplementary concept word, rare disease supplementary concept word, unique identifier, synonyms, population supplementary concept word, anatomy supplementary concept word]	24665
8	6 or 7	24665
9	5 and 8	9369
10	("unaccompanied minor*" or "accompanied minor*").mp. [mp=title, book title, abstract, original title, name of substance word, subject heading word, floating sub-heading word, keyword heading word, organism supplementary concept word, protocol supplementary concept word, rare disease supplementary concept word, unique identifier, synonyms, population supplementary concept word, anatomy supplementary concept word]	309
11	9 or 10	9509
12	exp mental health service/	72075
13	(("mental" or "psych*" or "behavio?r*" or "psychosocial") adj4 ("health care" or "service*" or "treatment*" or "support*" or "contact*")).mp. [mp=title, book title, abstract, original title, name of substance word, subject heading word, floating sub-heading word, keyword heading word, organism supplementary concept word, protocol supplementary concept word, rare disease supplementary concept word, unique identifier, synonyms, population supplementary concept word, anatomy supplementary concept word]	355055
14	12 or 13	355160
15	exp help seeking behavior/	17759
16	exp health care utilization/	718384

17	("help seeking" or "seek* help" or "treatment seek*" or "seek* treatment" or "help seeking behavio?r" or "utili?ation" or "use*" or "contact*").mp. [mp=title, book title, abstract, original title, name of substance word, subject heading word, floating sub-heading word, keyword heading word, organism supplementary concept word, protocol supplementary concept word, rare disease supplementary concept word, unique identifier, synonyms, population supplementary concept word, anatomy supplementary concept word]	13157937
18	15 or 16 or 17	13514972
19	(barrier* or hurdle or obstacle or obstruct* or refusal or imped* or promot* or facilitat* or support* or enabl* or cause* or reason*).mp. [mp=title, book title, abstract, original title, name of substance word, subject heading word, floating sub-heading word, keyword heading word, organism supplementary concept word, protocol supplementary concept word, rare disease supplementary concept word, unique identifier, synonyms, population supplementary concept word, anatomy supplementary concept word]	11182312
20	exp health care access/	140082
21	("access" or "affordability" or "availability" or "accessibility" or "accommodation" or "acceptability" or "stigma" or "awareness").mp. [mp=title, book title, abstract, original title, name of substance word, subject heading word, floating sub-heading word, keyword heading word, organism supplementary concept word, protocol supplementary concept word, rare disease supplementary concept word, unique identifier, synonyms, population supplementary concept word, anatomy supplementary concept word]	1697550
22	20 or 21	1714660
23	18 or 19 or 22	20742582
24	11 and 14 and 23	914
25	limit 24 to (english language and humans and yr="2014 -Current")	718

Global Health

	Search term	Results
1	children/	509022
2	adolescents/	111139
3	young adults/	25944
4	(young or youth or child* or adolescen* or teen*).mp. [mp=abstract, title, original title, heading words, cabicodes words]	744887
5	1 or 2 or 3 or 4	744887
6	refugees/	7879
7	(refugee* or "asylum seeker*" or "forced migrant*").mp. [mp=abstract, title, original title, heading words, cabicodes words]	6854
8	6 or 7	10150
9	5 and 8	3265
10	("unaccompanied minor*" or "accompanied minor*").mp. [mp=abstract, title, original title, heading words, cabicodes words]	79
11	9 or 10	3306
12	(health services or mental health).sh.	542364
13	(("mental" or "psych*" or "behavio?r*" or "psychosocial") adj4 ("health care" or "service*" or "treatment*" or "support*" or "contact*")).mp. [mp=abstract, title, original title, heading words, cabicodes words]	36570
14	12 or 13	555639
15	health care utilization/	23560
16	("help seeking" or "seek* help" or "treatment seek*" or "seek* treatment" or "help seeking behavio?r" or "utili?ation" or "use*" or "contact*").mp. [mp=abstract, title, original title, heading words, cabicodes words]	2134831
17	15 or 16	2134831
18	(barrier* or hurdle or obstacle or obstruct* or refusal or imped* or promot* or facilitat* or support* or enabl* or cause* or reason*).mp. [mp=abstract, title, original title, heading words, cabicodes words]	1534478
19	access/	23779
20	("access" or "affordability" or "availability" or "accessibility" or "accommodation" or "acceptability" or "stigma" or "awareness").mp. [mp=abstract, title, original title, heading words, cabicodes words]	343790
21	19 or 20	343790
22	17 or 18 or 21	3094507
23	11 and 14 and 22	1196
24	limit 23 to (english language and yr="2014 -Current")	941

PsycINFO

	Search term	Results
1	(young or youth or child* or adolescen* or teen* or "young adult*").mp. [mp=title, book title, abstract, original title, name of substance word, subject heading word, floating sub-heading word, keyword heading word, organism supplementary concept word, protocol supplementary concept word, rare disease supplementary concept word, unique identifier, synonyms, population supplementary concept word, anatomy supplementary concept word]	1467330
2	exp Refugees/	10097
3	(refugee* or "asylum seeker*" or "forced migrant*").mp. [mp=title, abstract, heading word, table of contents, key concepts, original title, tests & measures, mesh word	14879
4	2 or 3	14879
5	1 and 4	52485618
6	("unaccompanied minor*" or "accompanied minor*").mp. [mp=title, abstract, heading word, table of contents, key concepts, original title, tests & measures, mesh word]	263
7	5 or 6	5743
8	exp Mental Health Services/	85288
9	(("mental" or "psych*" or "behavio?r*" or "psychosocial") adj4 ("health care" or "service*" or "treatment*" or "support*" or "contact*")).mp. [mp=title, abstract, heading word, table of contents, key concepts, original title, tests & measures, mesh word]	305975
10	8 or 9	333289
11	exp Health Care Seeking Behavior/ or exp Help Seeking Behavior/ or exp Health Care Utilization/ (35072)	37044
12	("help seeking" or "seek* help" or "treatment seek*" or "seek* treatment" or "help seeking behavio?r" or "utili?ation" or "use*" or "contact*").mp. [mp=title, abstract, heading word, table of contents, key concepts, original title, tests & measures, mesh word]	2047541
13	11 or 12	2051027
14	exp Treatment Barriers/	8691
15	(barrier* or hurdle or obstacle or obstruct* or refusal or imped* or promot* or facilitat* or support* or enabl* or cause* or reason*).mp. [mp=title, abstract, heading word, table of contents, key concepts, original title, tests & measures, mesh word]	1767219
16	14 or 15	1767219
17	exp Health Care Access/	11954
18	("access" or "affordability" or "availability" or "accessibility" or "accommodation" or "acceptability" or "stigma" or "awareness").mp. [mp=title, abstract, heading word, table of contents, key concepts, original title, tests & measures, mesh word]	410477

19	17 or 18	414594
20	13 or 16 or 19	3206089
21	7 and 10 and 20	826
22	limit 21 to (human and english language and yr="2014 -Current")	489

The International Bibliography of the Social Sciences (IBSS)

	Search term	Results
1	((("young" OR "youth" OR "child*" OR "adolescen*" OR "teen*") NEAR/4 ("refugee*" OR ("asylum seeker" OR "asylum seekers") OR ("forced migrants")) OR (("unaccompanied minor" OR "unaccompanied minors") OR "accompanied minor*")) AND ((("mental" OR "psych*" OR "behavio?r*" OR "psychosocial") NEAR/4 ("health care" OR "healthcare" OR "service*" OR "treatment*" OR "support*" OR “contact*”)) AND (("help seeking" OR "seek* help" OR "treatment seek*" OR "seek* treatment" OR "help seeking behavio?r" OR "utili?ation" OR "use*" OR “contact*”) OR ("barrier*" OR "hurdle*" OR "obstacle*" OR "obstruct*" OR "refusal" OR "imped*" OR "promot*" OR "facilitat*" OR "support*" OR "enabl*" OR "cause*" OR "reason*") OR ("affordability" OR "availability" OR "accessibility" OR "accommodation" OR "acceptability" OR "stigma" OR "awareness")))) AND stype.exact("Scholarly Journals") AND la.exact("English") AND PEER(yes) AND pd	465

C. Quality appraisal

JBI Checklist for Cohort Studies

Question	Amin et al (2020)	Axelsson et al (2020)	Barghadouch et al (2016)	Berg et al (2020)	Betancourt et al (2017)	Björkenstam et al (2022)	de Montgomery et al (2020)	Fine et al (2022)	Gill et al (2017)	Gubi et al (2021)	Kamali et al (2023)	Kane et al (2014)	Karadag & Calisgan (2021)	Mohamud et al (2024)	Poyraz Findik et al (2021)	Saunders et al (2018a)	Saunders et al (2023)	Taipale et al (2020)
1. Were the two groups similar and recruited from the same population?	Y	Y	Y	Y	Y	Y	Y	N/A	Y	Y	Y	N/A	N/A	Y	Y	Y	Y	Y
2. Were the exposures measured similarly to assign people to both exposed and unexposed groups?	Y	Y	Y	Y	Y	Y	Y	N/A	Y	Y	Y	N/A	N/A	Y	Y	Y	Y	Y
3. Was the exposure measured in a valid and reliable way?	Y	Y	Y	Y	Y	Y	Y	Y	Y	Y	Y	Y	U	Y	Y	Y	Y	Y
4. Were confounding factors identified?	Y	Y	Y	Y	Y	Y	Y	Y	Y	Y	Y	Y	N	Y	Y	Y	Y	Y
5. Were strategies to deal with confounding factors stated?	Y	Y	Y	Y	N	Y	Y	N	Y	Y	Y	N	N	Y	U	Y	Y	Y
6. Were the groups/participants free of the outcome at the start of the study (or at the moment of exposure)?	Y	Y	Y	Y	U	Y	Y	U	Y	Y	U	U	U	U	U	U	U	Y
7. Were the outcomes measured in a valid and reliable way?	Y	Y	Y	Y	Y	Y	Y	Y	Y	Y	Y	Y	Y	Y	Y	Y	Y	Y
8. Was the follow up time reported and sufficient to be long enough for outcomes to occur?	Y	Y	Y	Y	Y	Y	Y	Y	Y	Y	Y	Y	U	Y	U	Y	Y	Y
9. Was follow up complete, and if not, were the reasons to loss to follow up described and explored?	Y	Y	Y	Y	N	Y	Y	Y	Y	Y	U	Y	U	U	U	Y	Y	Y
10. Were strategies to address incomplete follow up utilised?	U	U	U	U	N	U	U	U	U	U	U	U	U	U	U	U	U	U
11. Was appropriate statistical analysis used?	Y	Y	Y	Y	N	Y	Y	U	Y	Y	Y	U	N	Y	Y	Y	Y	Y
Y: Yes, N: No, U: Unclear, N/A: Not applicable																		

JBI Checklist for cross-sectional

Question	Mazumdar et al (2022)	Saunders et al (2018b)	Toulany et al (2023a)	Toulany et al (2023b)
1. Were the criteria for inclusion in the sample clearly defined?	Y	Y	Y	Y
2. Were the study subjects and the setting described in detail?	Y	Y	Y	Y
3. Was the exposure measured in a valid and reliable way?	Y	Y	Y	Y
4. Were objective, standard criteria used for measurement of the condition?	Y	Y	Y	Y
5. Were confounding factors identified?	Y	Y	U	U
6. Were strategies to deal with confounding factors stated?	Y	Y	U	U
7. Were the outcomes measured in a valid and reliable way?	Y	Y	Y	Y
8. Was appropriate statistical analysis used?	Y	Y	U	U
Y: Yes, N: No, U: Unclear, N/A: Not applicable				
