# Supplementary material for: A systematic review of global mental health service utilisation in young refugees and asylum seekers
Source: BJPsych Open. 2026 Mar 2;12(2):e76. doi: 10.1192/bjo.2025.10963 (PMC12963839; doi:10.1192/bjo.2025.10963)
Supplement: Abou Seif et al. supplementary material 2 — Abou Seif et al. supplementary material [file S2056472425109630sup002.pdf]

SUPPLEMENTARY TABLES

Supplementary Table 1. Cohort studies

Authors	Location	Population and sample size	Mental Health Outcome	Case determination	Service Use Outcome	Nature of Service	Key findings
Amin et al (2020) [34]	Sweden	Refugees (aged 16-25) from Eritrea, Ethiopia, Somalia, Afghanistan, Iran, Iraq, Syria, Chile and Former Yugoslavia<br><br>N=41,884	Depressive disorders, anxiety disorders, PTSD, and other stress-related disorders	ICD-10	First treatment for common mental disorders (CMDs) during the 7-year follow-up period.	Inpatient, outpatient, or prescription of antidepressant	13.6% of refugees had a first treatment for CMDs vs 15.9% of Swedish peers. Young refugees also had a 25% lower likelihood of being first CMD treatment (aHR: 0.75, 9% CI 0.73-0.77)<br><br>Stratified HRs were lower for refugees of all countries of origin except Iran (aHR: 1.15, 9% CI: 1.05-1.26). aHRs were particularly low for refugees from the Horn of Africa (aHR range: 0.43-0.54)
Axelsson et al (2020) [35]	Sweden	Unaccompanied refugee minors (aged <18)<br><br>N=6,133	Unspecified – all mental health diagnoses requiring mental health care included	Unspecified	First time use of any psychiatric services during the study period.	Inpatient, outpatient, or prescription of psychotropic drugs	Compared to Swedish youth, URM had higher aHRs for all psychiatric care use except ADHD medication.<br>• Inpatient care: 1.30 (95% CI: 1.10-1.45)<br>• Outpatient care: 1.10 (95% CI: 1.01-1.18)<br>• Prescribed psychotropic drugs: 1.10 (95% CI: 1.01-1.19)<br>• ADHD medication: 0.06 (95% CI: 0.04-0.10)<br>• Tranquilisers: 2.21(95% CI: 2.04-2.40)<br>• Neuroleptics: 1.27(95% CI: 1.04-1.56)<br>• Antidepressants: 1.58 (95% CI: 1.44-1.73)<br>• Sedatives: 3.25 (95% CI:3.01-3.51)

Authors	Location	Population and sample size	Mental Health Outcome	Case determination	Service Use Outcome	Nature of Service	Key findings
Barghadouch et al (2016) [36]	Denmark	Refugee (<18) children from Asia, The Middle East, Sub-Saharan Africa, and former Yugoslavia<br><br>N=24,427	Unspecified – all mental health diagnoses requiring mental health care included	Unspecified – assessed by mental health professionals	First-time psychiatric contact	Inpatient, outpatient, emergency room, or private services	3.5% of refugee children vs 7.7% Danes accessed psychiatric care.<br><br>Any first-time contact (aRRs):<br>• Boys: 0.42 (95% CI: 0.40 – 0.45)<br>• Girls: 0.35 (95% CI: 0.33-0.37)<br><br>Inpatient:<br>• Boys: 0.59 (95% CI: 0.43– 0.83)<br>• Refugee girls: 0.63 (95% CI: 0.47-0.85)<br><br>Outpatient:<br>• Boys: 0.47 (95% CI: 0.44 – 0.51)<br>• Girls: 0.42 (95% CI: 0.39-0.46)<br><br>ER:<br>• Boys: 0.71 (95% CI: 0.59 – 0.84)<br>• Girls: 0.83 (95% CI: 0.73-0.94)<br><br>Private:<br>• Boys: 0.25 (95% CI: 0.22 – 0.28)<br>• Girls: 0.16 (95% CI: 0.14-0.18)<br><br>RRs lower among refugees of all geographic origins (aRR range: 0.22-0.57), but particularly those from Sub-Saharan Africa (aRR range: 0.22-0.31).

Authors	Location	Population and sample size	Mental Health Outcome	Case determination	Service Use Outcome	Nature of Service	Key findings
Berg et al (2020)[37]	Sweden	Refugee children (aged 11-18)<br><br>Classified as either “asylum” or “family reunification” based on the grounds for their residence permit<br><br>N=18,831	ADHD, ASD, intellectual disability, OCD, anxiety, depression, severe stress including PTSD, phobic syndromes, eating disorders, “outacting”, bipolar disorder, schizophrenia, substance use	ICD-10	First visit to a child psychiatric service	Any psychiatric service	Compared to Swedish youth with two Swedish parents, refugee children from low- and middle-income countries had lower aHRs for psychiatric care.<br>• Foreign-born refugee from high-income country: 0.92 (95% CI: 0.70-1.21)<br>• Foreign-born refugee from middle-income country: 0.51 (95% CI: 0.46-0.56)<br>• Foreign-born refugee from high-income country: 0.34 (95% CI: 0.28-0.42)<br><br>Service use increased with residency duration, particularly for those who received residency on the grounds of asylum (aHR <5 years: 0.59, 95% CI 0.48-0.72; aHR 10+ years: 0.74, 95% CI 0.64-0.86) vs family reunification (aHR <5 years: 0.25, 95% CI 0.17-0.36; aHR 10+ years: 0.37, 95% CI 0.28-0.50)
Betancourt et al (2017)[33]	USA	Refugee children and adolescents<br><br>N=60	All DSM-4 disorders	DSM-4	Service use in 30 days prior to intake and most recent 30 days of care at a (NCTSN)	Inpatient, outpatient, Emergency room, or primary care	Refugees more likely than non-refugee immigrants to receive in-home counselling (11.8% vs 3.6%, p<0.05) and primary care (23.3% vs 5.8%, p<0.05).

Authors	Location	Population and sample size	Mental Health Outcome	Case determination	Service Use Outcome	Nature of Service	Key findings
Björkenstam et al (2022) [38]	Sweden	Refugees (unaccompanied and accompanied)<br><br>N=32,481<br><br>Unaccompanied: n = 2,896<br><br>Accompanied: n = 29,585	Substance abuse disorders, schizophrenia and other non-affective psychotic disorders, bipolar disorder, depressive disorders, anxiety disorders, reaction to severe stress and adjustment disorders including PTSD, PTSD as a separate category, personality disorders, autism spectrum disorders, behavioural and emotional disorders with onset occurring in childhood and adolescence, and ADHD as a separate category.	ICD-10	First psychiatric care utilisation	Inpatient or outpatient	Compared to Swedish-born youth, young refugees were significantly less likely to use psychiatric care for any mental disorder (aHR: 0.75, 95% CI: 0.72-0.77)<br><br>Refugees that arrived in Sweden unaccompanied were slightly less likely than those accompanied to use psychiatric care<br>• URM aHR: 0.65 (95% CI: 0.58-0.72)<br>• ARM aHR: 0.75 (95% CI: 0.72-0.78)<br><br>This was consistent across all mental disorders except for higher rates in schizophrenia, reaction to severe stress/adjustment disorders and PTSD.<br><br>Psychiatric care use was higher in those that had resided in Sweden for more than 10 years, and for those that arrived in Sweden <6 years of age.

Authors	Location	Population and sample size	Mental Health Outcome	Case determination	Service Use Outcome	Nature of Service	Key findings
de Montgomery et al (2020) [16]	Denmark	Refugees (aged 15-22)<br><br>N=13,027	Drug related, schizophrenia, affective, neurotic and stress related, developmental, and other disorders	ICD-10	Type and amount of first contact with psychiatric healthcare	Inpatient, outpatient, emergency room, private care, or psychotropic drug purchases	Refugee girls and boys were overall less likely than Danish peers to have a first contact for most mental health disorders (aORs: 0.34-0.65), except schizophrenia in boys (aORs: 0.92-2.13). Refugees had more inpatient and emergency room contacts, but less outpatient and private contacts, and prescribed medicine purchases.<br><br>Service use was particularly low for neurodevelopmental and drug-related disorders in refugee boys (neurodevelopmental aOR ranges: 0.03 – 0.15; drug-related aOR ranges: 0.29 – 0.7) and girls (neurodevelopmental aOR ranges: 0.11-0.16; drug-related aOR ranges: 0.23- 0.4).

Authors	Location	Population and sample size	Mental Health Outcome	Case determination	Service Use Outcome	Nature of Service	Key findings
Fine et al (2022) [46]	LMICs	Refugees (<5 years old)<br><br>Number of participants within age group not specified	Epilepsy/seizures, alcohol/substance use disorders, intellectual disability, psychotic disorders, severe emotional disorders (including depression and PTSD), medically unexplained somatic complaints, and other psychological complaints.	Mental, Neurological, and Substance Use (MNS) categories	MNS visits	Primary care MNS services	Across all categories, MNS service use rates per 1000 per month were lower among children under five compared to refugees aged five and older.<br><br>Across the study period, epilepsy/seizures had the highest visit rates, ranging from 0.44 (SD=0.71) to 0.59 (SD=0.99) in boys and 0.33 (SD=0.71) to 0.48 (SD=0.93) in girls. This was followed by visits for intellectual disabilities, ranging from from 0.04 (SD = 0.09) to 0.11 (SD = 0.27) in boys, and 0.02 (SD = 0.03) to 0.09 (SD = 0.29) in girls.
Gill et al (2017) [39]	Canada	Refugees (aged 10-24)<br><br>N=2,194	Diagnoses included in ICD-10	ICD-10	First contact mental health ED visit	Emergency department	Compared with non-immigrants, refugee immigrants had a greater likelihood of first contact in ED for mental health (Risk Ratio = 1.17, 95% CI: 1.13-1.21)

Authors	Location	Population and sample size	Mental Health Outcome	Case determination	Service Use Outcome	Nature of Service	Key findings
Gubi et al (2021)[17]	Sweden	Refugees<br><br>N=3,151<br><br>Unaccompanied n=1,277<br><br>Accompanied: N=1,874	Diagnoses included in ICD-10	ICD-10	First contact with any mental health services	Inpatient, outpatient, primary care, or prescription of psychotropic drug	Refugee youth aged 15-20 had lower use compared to Swedish youth (HR: 0.64, 95% CI: 0.57 – 0.73). No significant differences found in refugees aged 0-10<br><br>URMs had higher use in their first two years in Sweden (OR: 3.39, 95% CI: 2.96 – 3.85). ARMs also had higher use, but not to the same extent (OR: 1.53, 95% CI: 1.31-1.79). After approximately six years, service use for both became significantly lower than Swedish counterparts.<br><br>Lower rates of service were partly explained by decreased diagnosis of neurodevelopmental conditions in refugees, with HRs being significantly lower than Swedish peers across all age groups except 0-5 years.
Kamali et al (2023)[42]	Canada	Refugees (aged 4-17)<br><br>N=573	Internalising and externalising behaviours	2014 OCHS-Emotional Behavioural Scales (OCHS-EBS)	Mental health-related service contact in the past 6-months	Outpatient, emergency room, primary care, school-based, other (hotline, spiritual leader, alternative health practitioner)	Compared to non-immigrant counterparts, refugee youth were less likely to have mental health-related service contacts (aOR: 0.64, 95% CI: 0.58-0.71)

Authors	Location	Population and sample size	Mental Health Outcome	Case determination	Service Use Outcome	Nature of Service	Key findings
Kane et al (2014) [45]	LMICs	Refugees<br><br>Number of participants within age group not specified	Epilepsy/seizures, alcohol/substance use disorders, intellectual disability, psychotic disorders, severe emotional disorders (including depression and PTSD), medically unexplained somatic complaints, and other psychological complaints.	Mental, Neurological, and Substance Use (MNS) categories	MNS visits from	MNS services	MNS visit rates were lower among children younger than five compared to those five and older across all MSN categories.<br><br>Overall MNS rates were higher in boys (mean: 1.13, SD: 1.75) in comparison to girls (mean:0.80, SD: 1.20)<br><br>Epilepsy/seizure accounted for the greatest proportion of MNS visits in both boys (82.7%) and girls (82.3%).<br><br>Intellectual disability accounted for the second highest proportion of MNS visits in both boys (10.6%) and girls (9.0%).
Karadag & Calisgan (2021) [47]	Turkey	Syrian refugees (aged 0-18)<br><br>N=400	Intellectual disability, ASD, ADHD, conduct disorder, specific LD, motor disorders, schizophrenia and other psychotic disorders, depressive disorders, anxiety disorders, OCD and related disorders, trauma and stressor related disorders, elimination disorders, sleep-wake disorders	DSM-5	Presentation for psychiatric treatment	Outpatient	Refugees were most likely to present to psychiatric treatment for intellectual disability (19%), trauma and stressor related disorders (17%), and ADHD (12%).<br><br>Half the sample (50%) were not initiated on any medication. Only 16% were prescribed SSRIs and 17% were prescribed antipsychotics.

Authors	Location	Population and sample size	Mental Health Outcome	Case determination	Service Use Outcome	Nature of Service	Key findings
Mohamud et al (2024) [43]	Canada	Refugees (aged 3-17) in Ontario, Canada<br><br>N=2,529	Unspecified	Unspecified	Number of mental healthcare visits	Outpatient, primary care	Young refugees were significantly less likely to use virtual mental health visits compared to economic class immigrants, regardless of Canadian language ability. However, refugees with non-Canadian language ability had 9% lower risk of virtual care utilisation compared to refugees with Canadian language ability.
Poyraz Fındık et al (2021) [44]	Turkey	Syrian refugee children (aged 0-18)<br><br>N=91	ADHD, ODD, ASD, global developmental delay and intellectual disabilities, specific learning disabilities, major depressive disorder, GAD, separation anxiety disorder, PTSD, enuresis, language disorders	DSM-5	Mental healthcare visits	Outpatient	Refugee children attended significantly fewer appointments for mental healthcare than non-refugee children ($p<0.05$).<br><br>Most common diagnoses for refugee children were ADHD (36.3% vs 47.6% in non refugees), depression (28.6% vs 10% in non-refugees), and PTSD (22% vs 0% in non-refugees).<br><br>Proportion of refugee children that were free of any psychiatric diagnosis was 7x that of nonrefugees (8.8% vs 1.2%).

Authors	Location	Population and sample size	Mental Health Outcome	Case determination	Service Use Outcome	Nature of Service	Key findings
Saunders et al (2018a) [15]	Canada	Young refugees (aged 10-24)<br><br>1996-1998: N=284,150 (17.7% refugees)<br><br>1999-2001: N=304,991 (17.7% refugees)<br><br>2002-2004: N=322,173 (17.9% refugees)<br><br>2005-2007: N=337,163 (17.7% refugees)<br><br>2008-2010: N=326,556 (18% refugees)<br><br>2011-2012: N=265,061 (18.7% refugees)	ICD mental health disorders, or a secondary diagnosis of self-inflicted injury	ICD-9 and ICD-10	Mental health service utilisation between 1996-2012 in 2-year intervals.	Inpatient, outpatient, or emergency department	Compared to non-refugee immigrants, refugees had higher aRRs for ED visits and hospitalisations. Refugee RRs for outpatient visits were slightly lower than majority peers<br><br>Hospitalisations:<br>-Refugees (0-5 years residence): aRR 1.02 (95% CI 0.97-1.08),<br>-Refugees (5-10 years residence): aRR 1.12 (95% CI 1.03-1.21).<br><br>ED visits:<br>-Refugees (0-5 years residence): aRR 1.14 (95% CI 1.07-1.22)<br>-Refugees (5-10 years residence): aRR 1.11 (95% CI 1.02-1.20)<br><br>Outpatient physician visits:<br>-Refugees (0-5 years residence): aRR 0.95 (95% CI 0.93-0.96)<br>-Refugees (5-10 years residence): aRR 0.95 (95% CI 0.93-0.97).

Authors	Location	Population and sample size	Mental Health Outcome	Case determination	Service Use Outcome	Nature of Service	Key findings
Saunders et al (2023) [40]	Canada	Refugees (aged 3-17)<br><br>N=23,287	Unspecified	Unspecified	Health services utilisation	Inpatient, outpatient, or emergency	In comparison to matched Ontario-born youth, refugees had less outpatient (10.5% vs 11.3%) and ED visits (0.3% vs 0.7%) for mental health than Ontario-born, but similar rates for hospitalisations (0.1% vs 0.2%).
Taipale et al (2021) [41]	Sweden	Refugees (aged 16-25)<br><br>Antidepressant cohort N=3,936<br><br>Other pharmacotherapy cohort N=4,506	Common mental health disorders (CMDs) defined as major depressive disorder and anxiety disorder, PTSD	ICD-10	Use of psychotropic medication	Antidepressants, anxiolytics, mood stabilisers, or antipsychotics	Refugees less likely to initiate antidepressant use compared to Swedish youth (40.5% vs 59.6%, aOR: 0.43, 95% CI: 0.39-0.48).<br>• <5 years of residency associated with decreased initiation (OR 0.76, 95%CI: 0.63-0.92).<br>• Older age associated with increased initiation (OR 1.07, 95% CI: 1.04-1.10)<br>• Those born in Somalia (OR 0.70, 95% CI: 0.49-0.99) and Iraq (OR 7.95, 95% CI: 0.63-0.99) were less likely to initiate antidepressant<br><br>Other medication was also initiated less in young refugees (51.5% vs 61.8%).<br>• Anxiolytics were less common (52.3% vs 60.0%)<br>• Hypnotics were more common (38.4% vs 32.8%)

Abbreviations:
- **aHR** = adjusted hazard ratio
- **aOR** = adjusted odds ratio
- **aRR** = adjusted rate ratio

Table 2. Cross-sectional studies

Authors	Location	Population and sample size	Mental Health Outcome	Case determination	Service Use Outcome	Nature of Service	Key findings
Mazumdar et al (2022) [48]	Australia	Refugees (aged 0-24)<br><br>Number of young refugees not specified	Psychotic disorders, reaction to severe stress and adjustment disorders, depressive episode, other anxiety disorders, bipolar affective disorders, schizophrenia	ICD-10	Service contacts	Outpatient	Refugees aged 0-17 (SR 0.41, 95% CI: 0.39-0.45) and 18-24 (SR 0.10, 95% CI: 0.09-0.11) were less likely to use mental health services when compared to Australian youth.
Saunders et al (2018b) [49]	Canada	Refugees (aged 10-24)<br><br>N=2,194	Acute stress, anxiety, mood or affective disorder, residual self-harm, psychotic disorders, substance-related disorders, other	ICD-10	First time presentation to ED for mental health condition	Emergency department	Young refugees had a higher proportion of first mental health contact in the ED (61.3%) in comparison to non-refugee immigrant youth (57.6%) and non-immigrant youth (51.3%)<br><br>Service use in young refugees was significantly higher than in non-immigrant youth (aRR: 1.17, 95% CI: 1.13-1.21)<br><br>The most common diagnoses at ED visits for refugees were substance-related disorders (74.5% vs 66.8%), residual self-harm (69.8% vs 50.8%), acute stress (62.1% vs 47.5%), and anxiety (57.3% vs 50.4%).

Authors	Location	Population and sample size	Mental Health Outcome	Case determination	Service Use Outcome	Nature of Service	Key findings
Toulany et al (2023a) [50]	Canada	Refugees (aged 3-17)<br><br>N=26,346	Psychotic disorders, mood and anxiety disorders, substance use disorders, social problems, and neurodevelopmental and other concerns	ICD-10	Monthly outpatient mental health-related visits during the COVID-19 pandemic	Outpatient, primary care	Refugee children who were new to mental healthcare had 20% higher than expected mental health visits rates during the pandemic (aRR: 1.20, 95% CI: 1.11-1.28).<br><br>There was no significant change in mental healthcare use in refugee children with continuing mental health care needs.
Toulany et al (2023b) [51]	Canada	Refugees (aged 3-17)<br><br>N=26,346	Disorders specified in the ICD-10	ICD-10	Number of mental health-related visits pre- and during the COVID-19 pandemic	Outpatient	No significant changes in refugee use of physician-based outpatient mental-health related visits pre- vs during COVID pandemic.<br><br>In comparison to non-immigrants, refugees had lower observed and expected mental health-related visit rates.<br>• Refugees: 3.6 and 3.7 per 1,000 population respectively<br>• Non-immigrants: 7.2 and 7.1 per 1,000 population respectively

Abbreviations:
- **SR** = standardised ratio
- **aRR** = adjusted rate ratio
